# Computed tomography of the paranasal sinuses in the preoperative evaluation of candidates for endonasal surgery

**DOI:** 10.1590/0100-3984.2019.52.3e3

**Published:** 2019

**Authors:** Eloisa Maria Santiago Gebrim

**Affiliations:** 1 Director of the Head and Neck Imaging and Computed Tomography at the Instituto de Radiologia do Hospital das Clínicas da Faculdade de Medicina da Universidade de São Paulo (InRad/HC-FMUSP), Director of the Head and Neck Imaging at Hospital Sírio-Libanês, São Paulo, SP, Brazil. E-mail: eloisa.gebrim@hc.fm.usp.br.

Computed tomography (CT) is the imaging method of choice for the evaluation of patients with chronic rhinitis, especially those who will undergo endonasal surgery. For this procedure to be performed safely, avoiding iatrogenic injury, it is essential that the surgeon has an accurate map of the bone structures and drainage pathways of the paranasal sinuses, as well as their anatomic variations, especially those that can result in complications or failure of the procedure^(^^1^^)^. However, important information regarding the anatomy of the paranasal sinuses is often omitted from the radiological report.

One question that radiologists should always ask is whether their examination report provides the necessary information to the requesting physician, especially when they are reporting the findings of preoperative examinations. Reports of CT scans of the paranasal sinuses often fail to provide that necessary information.

A survey of otolaryngologists in Canada showed that the majority (67%) would like to have more relevant information in reports of CT scans of the paranasal sinuses. According to that study, such reports omit much of the information that is essential to the preoperative planning process^(^^2^^)^.

Various studies have provided a review of the anatomy of the paranasal sinuses^(^^3^^-^^5^^)^, reporting variations that can significantly reduce the chance that the endonasal procedure will be successful, proposing models of structured reports or checklists of items that should be included in the report of a CT scan of the paranasal sinuses^(^^5^^,^^6^^)^.

O'Brien et al.^(^^6^^)^ suggested the use of the term "CLOSE"-signifying **C**ribriform plate, **L**amina papyracea, **O**nodi cell, **S**phenoid sinus pneumatization, and (anterior) **E**thmoidal artery-as a mnemonic to recall critics variants that should be mentioned in the report of a CT scan of the paranasal sinuses. The articles cited above underscore the importance of efficient communication between radiologists and surgeons, in order to improve the quality of reporting.

The creation of structured reports and contextual reports has been proposed for the various subspecialties of radiology, including neuroradiology^(^^7^^)^. Structured reports have advantages over free-text narrative reports in that they ensure the uniformity and clarity of the information reported, increasing its quality and facilitating its use in searches of the scientific literature^(^^8^^)^. Contextual reports, which are variations of the structured report, comprise models of reports for different clinical contexts. Thus, the radiologist will provide important information for a given disease or clinical situation, such as a preoperative evaluation. Mamlouk et al.^(^^7^^)^ proposed models of contextual reports for different clinical situations in neuroradiology, such as the staging of head and neck tumors and the evaluation of patients with multiple sclerosis, as well as the preoperative evaluation of the pituitary gland and of the paranasal sinuses.The contextual report for the preoperative evaluation of candidates for endonasal surgery proposed by those authors includes a description of the findings of mucosal thickening in the paranasal sinuses and lists the different anatomic variations that should be cited^(^^7^^)^.

In the previous issue of **Radiologia Brasileira**, Niemeyer et al.^(^^9^^)^ conducted a review of the anatomy of the paranasal sinuses. The authors emphasized the importance of the CT examination in the evaluation of the paranasal sinuses and their anatomic variations, which provides fundamental information for diagnosis and surgical planning.

Recent advances in endonasal surgery, which have allowed better access to the anterior, middle, and posterior cranial fossae, make it imperative to perform imaging tests, including CT, CT angiography, and magnetic resonance imaging, in the preoperative evaluation of surgical candidates. That expands the role of the radiologist, who must have knowledge of the complex anatomy of the paranasal sinuses in order to provide the information that will be important in the surgical planning^(^^10^^)^.
